# Characterisation of monoclonal antibodies specific for hamster leukocyte differentiation molecules

**DOI:** 10.1016/j.vetimm.2016.12.003

**Published:** 2017-01

**Authors:** Jennifer Rees, David Haig, Victoria Mack, William C Davis

**Affiliations:** a School of Veterinary Medicine and Science, University of Nottingham, Sutton, Bonington, LE12 5RD, UK; b Department of Veterinary Microbiology & Pathology, Washington State University, Pullman, WA, 99164-7040, USA

**Keywords:** Hamster, Monoclonal antibody, Flow cytometry, CD4, CD8

## Abstract

Flow cytometry was used to identify mAbs that recognize conserved epitopes on hamster leukocyte differentiation molecules (hLDM) and also to characterize mAbs developed against hLDM. Initial screening of mAbs developed against LDMs in other species yielded mAbs specific for the major histocompatibility (MHC) II molecule, CD4 and CD18. Screening of sets of mAbs developed against hLDM yielded 22 new mAbs, including additional mAbs to MHC II molecules and mAbs that recognize LDMs expressed on all leukocytes, granulocytes, all lymphocytes, all T cells, a subset of T cells, or on all B cells. Based on comparison of the pattern of expression of LDMs expressed on all hamster leukocytes with the patterns of expression of known LDMs in other species, as detected by flow cytometry (FC), four mAbs are predicted to recognize CD11a, CD44, and CD45. Cross comparison of mAbs specific for a subset of hamster T cells with a cross reactive mAb known to recognize CD4 in mice and one recognising CD8 revealed they recognize CD4. The characterization of these mAbs expands opportunities to use hamsters as an additional model species to investigate the mechanisms of immunopathogenesis of infectious diseases.

## 1 Introduction

The golden or Syrian hamster (*Mesocricetus auratus*) is used in biomedical research as a model for human and other animal diseases where the mouse is not appropriate. It is used as a model in multiple infectious diseases studies including Nipah virus (Wong et al., 2003), Hanta virus (Hammerbeck and Hooper, 2011), *Clostridium difficile* (Goulding et al., 2009) and safety testing of leptospirosis vaccines (Haake, 2006) and reviewed in Golden et al., 2015a, Golden et al., 2015b. Of particular interest to us is its usefulness as a small animal model for research into malignant catarrhal fever in ruminants (Buxton et al., 1988, Jacoby et al., 1988, Russell et al., 2009). Hamsters offer an opportunity for adoptive cell transfer experiments to explore pathogenesis, as they are highly inbred (Campbell et al., 1996). This may be attributable to the current lineage being derived from three siblings caught in 1930 limiting genetic heterogeneity and functionality (Phillips et al., 1981).

The usefulness of the hamster as a small animal model for biomedical research has been constrained by a lack of immunological reagents to detect LDM differentially expressed on lymphoid cell subsets. Of the few monoclonal antibodies (mAbs) specific for hamster leukocyte differentiation molecules (hLDM) that have been developed, most are no longer available (Liu et al., 1991, Witte et al., 1985, Witte and Streilein, 1983a, Witte and Streilein, 1983b, Witte and Streilein, 1986). More recently the Washington State University Monoclonal Antibody Centre has addressed the growing need for reagents for use with this species. The reagents developed thus far have only been partially characterized.

The objective of the study presented here has been to complete the initial characterization of mAbs produced by the Centre and screen a selected set of commercially available mAbs for cross reactivity with hLDMs. These mAbs are available to the research community for further detailed characterisation.

## 2 Materials and methods

### 2.1 Animals

Spleen, lymph node and blood from disease-free Syrian hamsters of variable age and either sex were obtained from Harlan Laboratories (Loughborough, U.K.) and Charles River Laboratories, (San Diego, CA). Additional animals were obtained from a breeding-colony maintained at WSU. Ethical approval for the work was obtained from site ethical review committees at both WSU and the SVMS, University of Nottingham. The Nottingham ethical review was performed by the local animal welfare and ethical review body (AWERB) and the work performed under ASPA (UK) project license 3003214 belonging to D. Haig.

### 2.2 Antibodies used in this study

The antibodies used in this study are shown in Table 1. The mAbs were developed from mice immunized with hamster peripheral blood leukocytes (HAB), thymocytes (HAT), lymph node mononuclear cells (HAL), or a mixture of non-adherent and adherent mononuclear splenocytes (HASA) (Davis et al., 1987, McNees et al., 2009). Additional mAbs screened for cross reactivity to hLDMs were from commercial sources and the WSU Monoclonal Antibody Centre http://vmp.vetmed.wsu.edu/resources/monoclonal-antibody-center

### 2.3 Tissue collection and preparation

Blood was collected into 10% lithium heparin or acid citrate dextrose (ACD). Spleen (Spln) and mesenteric lymph nodes (MLN) were removed and placed into PBS. Mononuclear cell suspensions were prepared by either lymphoprep (Nycomed, Pharmacia, Oslo, Norway), or ammonium chloride − potassium cell lysis buffer (ACK, Gibco Life Sciences, U.K.), which retains both MNC and granulocytes. To obtain enough cells for each experiment, spleen and MLN MNCs were pooled.

### 2.4 Flow cytometry

Two methods were used to process cells for flow cytometry. Blood was collected in acid citrate dextrose (ACD) and used at 50 μl with 50 μl of mAb in tissue culture medium or in ascites (15 μg/ml) in 15 ml centrifuge tubes. Following 15 min of incubation on ice, the cells were sedimented by centrifugation and re-suspended in 10 ml of PBS containing 0.5% horse serum (PBSh). Following removal of the PBSh, the cells were labelled with R-phycoerythrin (PE) or fluorescein conjugated isotype specific second step goat anti-mouse IgG1, IgG2a, IgG2b, IgG3 or IgM antibody (Invitrogen, Carlsbad, CA, USA) alone or in combination to determine specificity. The rbc were lysed with Becton Dickinson fix/lyse solution (BD, Oxford, UK) re-sedimented and then re-suspended in 2% paraformaldehyde in PBS. When using the cross reactive mAbs that recognize epitopes conserved on hamster CD4 and CD8 T cells, a fluorescein conjugated goat mouse-absorbed anti-rat IgG second step was used for rat GK1.5 IgG2b mAb. A PE conjugated 341 IgG1 was used with HAB1A IgG1 labelled with fluorescein conjugated anti-IgG1 Zenon reagent (Invitrogen, Carlsbad, CA, USA).

For the second method, cells in RPMI-1640 with 2% FBS or PBS with 2% FBS were distributed into 96 well culture plates (2 × 10^5^ to 1 × 10^6^ cells per well). 50 μl of appropriately diluted primary antibodies were added to the cells. Following incubation for 30 min (4 °C) the plates were centrifuged at 2000 rpm for 2 min and the supernatant removed. The cells were washed twice and incubated alone or in combination with isotype specific goat anti-mouse IgG or goat anti-rat IgG antibodies conjugated with fluorescein, PE or allophycocyanine (APC). In some experiments whole blood was incubated with mAbs and then the rbc were removed using 1 x BD FACSTM lysing solution before continuing with the labelling process. A Becton Dickinson FACS Calibur (Immunocytometry Systems, San Jose, CA, USA) (WSU) and Beckman Coulter EPICS Altra, FC500 and MoFlo XDP (School of Molecular Medical Sciences in the Queen’s Medical Centre, Nottingham, UK) were used to collect data. Data were analysed with FCS Express, LA, USA and the Beckman Coulter programs.

**Fig. 1:**
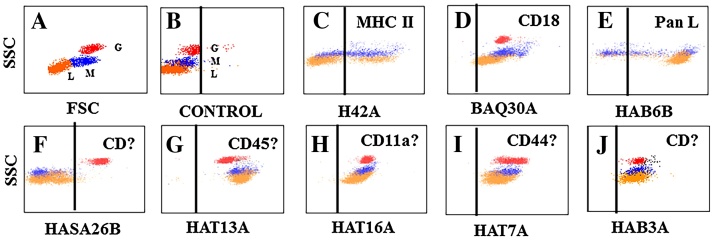
Hamster blood leukocytes. (Fig. 1A) The major populations of cells were visualized by side vs forward light scatter, dot plot and colour coded for cell subsets: orange = lymphocytes (L), blue = monocytes (M), red = granulocytes (G). It should be noted that gating for monocytes may include large lymphocytes. There is no distinct border separating lymphocytes from monocytes. (Fig. 1B) Example of cells incubated with a mixture of anti-IgG1, IgG2a, and IgG2b 2nd step reagents alone to show there was no background attributed to nonspecific labelling and the relative position of colour coded granulocytes, monocytes, and lymphocytes visualized in side scatter vs fluorescence. (Fig. 1C) Typical pattern of labelling with mAbs specific for MHC II cross species for humans, cattle, goats, sheep, and llama/alpaca. (Fig. 1D) Typical pattern of labelling with mAbs specific for CD18 cross species for human, cattle, goats, sheep, llama/alpaca, horse, dogs, and cats. (Fig. 1E) Unique pattern of expression of a mAb-defined molecule on all lymphocytes and apparent expression on a subset of monocytes. (Fig. 1F) Expression of a mAb-defined molecule on granulocytes (Background labelling of lymphocytes is attributable to cross reactive anti-IgM antibody present in the 2nd step reagent used in these studies). (Fig. 1G) Typical pattern of labelling with mAbs specific for CD45 cross species in humans, cattle, goats, sheep, llama/alpaca, rabbit. (Fig. 1H) Pattern of labelling similar to CD11a cross species in humans, cattle, goats, sheep, rabbit. (Fig. 1I) Pattern of labelling similar to CD44 cross species in humans, cattle, goats, sheep, horse, rabbits. (Fig, 1J) Pattern of labelling with no apparent match to known LDMs. It should be noted that multiple hamsters were used at WSU during the past 28 years to develop and characterize the mAbs described in this report. On some occasions, only one hamster was used to obtain some of the information presented here and on other occasions, multiple hamsters were used to pool blood for analysis. The best representative flow cytometric profiles were selected from different data sets for presentation here. (For interpretation of the references to color in this figure legend, the reader is referred to the web version of this article.)

For flow cytometric analysis (FC), three electronic gates were used to identify and colour code regions of a dot plot display in side (SSC) vs forward light scatter (FSC) containing lymphocytes (L, orange), predominantly monocytes (M, blue), and granulocytes (G, red) to track the different cell populations in SSC vs FL and in 2 colour combinations (Fig. 1 A and B) (Allen et al., 2009).

## 3 Results and discussion

**Fig. 2:**
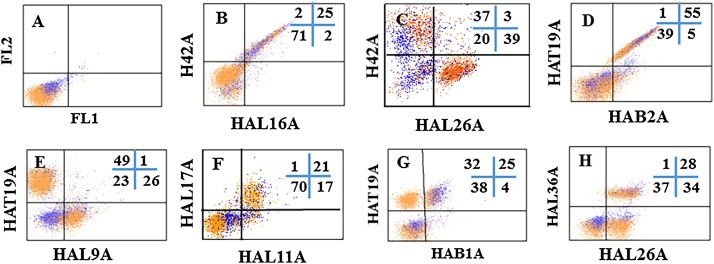
Hamster blood leukocytes after 2 colour labelling. (Fig. 2A) Representative plot FL-2 vs FL-1 of cells incubated with 2nd steps alone to show there was no background labelling with anti-IgG1, IgG2a 2nd step reagents. (Fig. 2B) Comparison of H42A (specific for MHC II) with HAL16A yielded a diagonal pattern of labelling showing HAL16A recognizes MHC II. (Fig. 2C) Comparison of labelling H42A with HAL26A showed HAL26A recognized a molecule not expressed on monocytes or B cells, indicating it recognized a molecule expressed on T cells. (Fig. 2D) Comparison of HA19A with HAB2A yielded a diagonal pattern of labelling indicating both mAbs recognized the same molecule expressed on T cells. (Fig. 2E) Comparison of HAT19A with HAL9A showed HAL9A recognizes a molecule not expressed on T cells or monocytes, inferring it recognizes a molecule expressed on B cells. (Fig. 2F) Comparison of labelling of HAL17A with HAL11A showed a pattern of labelling indicating they recognized different molecules on B cells, i.e., the pattern of labelling was diffuse, indicating the density of the molecules differed. (Fig. 2G) Comparison of HAT19A with HAB1A showed HAB1A recognized a subset of T cells. (Fig. 2H) A comparison of labelling HAL26A with HAL36A yielded a similar pattern of labelling, suggesting HAB1A and HAL36A recognized the same molecule. Two colour labelling showed HAB1A blocks co-labelling with HAL36A providing further evidence both of the mAbs recognized the same molecule. Two colour labelling was performed multiple times to verify specificity. Multiple hamsters were used to collect the data and to obtain the best representative FC profile.

Flow cytometry was used to screen for mAbs that recognize conserved epitopes expressed on hLDM (Saalmüller et al., 2005). Two ruminant cell-specific mAbs recognized conserved epitopes on hamster MHC II (H42A) (Davis et al., 1987) and CD18 (BAQ30A) (Naessens and Howard, 1993) (Table 1 and Fig. 1C and D respectively). As shown in Figs. 1 and 2 and summarized in Table 1, mAbs could be grouped and clustered according to unique patterns of expression of the mAb-defined molecules. By comparing their patterns of expression with that of H42A (MHC II, expressed on B cells and monocytes) it was possible to distinguish mAbs specific for MHC II, T cells and B cells. MAbs specific for MHC II yielded a diagonal pattern of expression, indicating the mAbs recognized epitopes on the same molecule (e.g. HAL16A, Fig. 2 B). MAbs specific for T cells distinguished populations of MHC II negative cells (e.g. HAL26A, Fig. 2 C) whereas mAbs specific for B cells distinguished a population cells negative for T cells and monocytes (e.g. HAT19A vs HAL9A Fig. 2 E). Comparison of the mAbs under study showed 4 mAbs recognized T cells (Table 1). Cross comparison of the mAbs showed they all recognized the same molecule, as evidenced by a diagonal pattern of labelling (e.g. HAT19A and HAB2A, Fig. 2 D). Two mAbs recognized a molecule expressed on a subset of T cells (HAB1A and HAL36A, Figs. 2 G and H). Cross comparison of the mAbs showed they recognized the same molecule. HAB1A blocked co-labelling with HAL36A. Comparison of the mAbs specific for T cells and B cells verified they were expressed on mutually exclusive populations (e.g. compare HAL9A and HAT19A, Fig. 2 E). Further comparison of the mAbs showed 5 mAbs recognized B cells (Table 1). Cross comparison of the mAbs specific for B cells showed they recognized 2 or more different molecules, as exemplified by the pattern of labelling obtained in the comparisons. MAbs recognizing different epitopes on the same population of cells exhibit a diffuse pattern of labelling (e.g. HAL17A and HAL11A Fig. 2F). Analysis of the remaining mAbs showed 2 mAbs recognized a molecule expressed on all lymphocytes and a subset of monocytes (HASA18A and HAB6B, Table 1 and Fig. 1 E). One mAb (HASA26B) recognized a molecule expressed on granulocytes (Fig. 1 F). Additional mAbs detected molecules expressed on all leukocytes, with specificities and patterns of expression characteristic of known LDM. Comparative studies conducted as part of international workshops during the 1990s and 2004 and during development of mAbs for use in llama/alpacas and rabbits, demonstrated patterns of expression of some LDMs, as detected by FC analysis using SSC vs fluorescence, were identical thus providing a way to predict the specificity of mAbs under study (Davis et al., 1995, Davis et al., 2007, Davis and Hamilton, 2008, Davis et al., 2000, Saalmüller et al., 2005, Tavernor et al., 1993, Tumas et al., 1994). Two mAbs recognized a molecule with expression characteristic of CD45 in multiple species (HASA25A and HAT13A (Table 1 and Fig. 1 G)), one characteristic of CD11a (HAT16A, Fig. 1 H), and one characteristic of CD44 (HAT7A, Fig. 1 I). The specificity of one mAb (HAB3A) could not be predicted based on the pattern of expression of the molecule (Fig. 1 J).

**Fig. 3:**
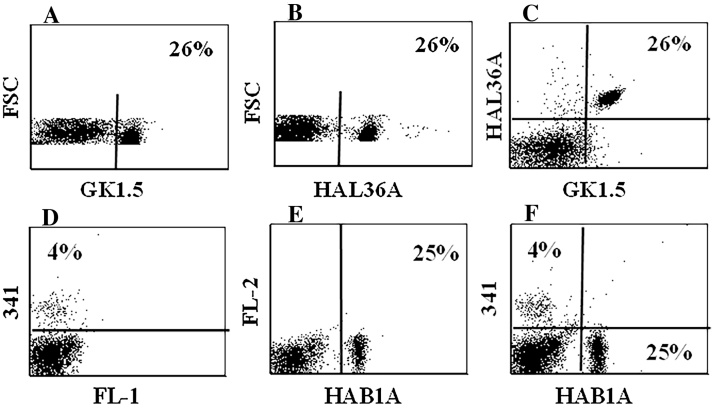
Cross reactive mAbs were used to validate specificity of HAB1A and HAL36A, a rat anti-mouse CD4 (GK1.5 IgG2b, (Dialynas et al., 1983)) cross reactive with hamster CD4 and mouse anti-rat CD8β 341 (IgG2a) specific for mouse CD8 (Hammerbeck and Hooper, 2011) showed HAB1A and HA36A recognize the same molecule (labelling only shown for HAL36A, Fig. 3A). Comparison with mAb 341 (mouse anti-rat CD8β) showed HAB1A labels a subset distinct from CD8 (Fig. 3B). Multiple hamsters were used in the UK and the US while validating cross reactivity and specificity.

Screening of commercial mAbs yielded a mAb made in rats against mouse CD4 that recognizes a conserved epitope expressed on hamster CD4 [eBioscience GK1.5 IgG2b (Dialynas et al., 1983)] and a mouse anti-rat CD8β mAb that recognized a conserved epitope on hamster CD8β [Biolegend 341 IgG1 (Hammerbeck and Hooper, 2011)]. Comparison of labelling of GK1.5 and with HAL36A (Fig. 3A) and 341 with HAB1A (Fig. 3B) showed both HAL36A and HAB1A recognized hamster CD4.

. Cross comparison with the anti-CD4 mAbs verified mAb 341 cross reacts with hamster CD8, filling in a gap in the reagents developed for use in hamsters (Fig. 3 D– F).

The development and availability of these 22 new mAbs, along with the commercial mAb number 341 that recognises hamster CD8, greatly expands opportunities to use hamsters in infectious disease research. In conclusion, the characterisation of mAbs to hamster hLDMs has provided further detail on specificity, making the mAbs more useful for the research community. These are now available for further characterisation and use by the research community.

## Conflict of interest

The authors declare no conflict of interest.

## Figures and Tables

**Table 1:** Monoclonal antibodies (WSU Monoclonal Antibody Centre) and Specificities.

mAb	Ig isotype	Putative specificity^1^	Specificity and% of cells^2^
H42A	IgG2a	MHC II	MHC II, 48%
BAQ30A	IgG1	CD18	CD18, 100%
HAL4A	IgG3	MHC class II	MHC class II, 50%
HAL16A	IgG1	MHC class II	MHC class II, 50%
HAB2A	IgG1	T	33%–43% (CD4 included)
HAL26A	IgG1	T	42%–63% (CD4 included)
HAT19A	G2a	T	39–53% (CD4 included)
HAT24A	IgG1	T	53%–73%
HAB1A	IgG1	T subpopulation	12–44% (CD4)
HAL36A	IgG2a	T subpopulation	16–42% (CD4)
HAL9A	IgG1	B	n.d.
HAL11A	IgG1	B	n.d.
HAL14A	IgG2b	B	B 23%
HAL17A	IgG2a	B	n.d.
HASA7A	IgG1	B	34–49% not CD4
HAB6B	IgG2a	Pan lymphocyte (+monocyte subset?)	n.d.
HASA18A	IgM	Pan lymphocyte (+monocyte subset?)	n.d.
HASA25A	IgG1	CD45 predicted	n.d.
HAT13A	IgG2b	CD45 predicted	CD45
HAT7A	IgG2a	CD44 predicted	n.d.
HAT16A	IgG2b	CD11a predicted	n.d.
HAB3A	IgG1	Pan leukocyte	>95% incl CD4 and CD?
HASA26B	IgG1	Granulocyte +	n.d.

Legend:

1 Based on labelling characteristics of lymphocyte, monocyte and granulocyte-enriched fractions of whole blood leukocytes and 2-colour FC comparison with MHC class II positive and negative fractions of the leukocytes.

2 Proposed specificities based on two colour comparisons of MNC (PBMC or spleen/MLN MNC) labelling by the mAbs compared to each other and a defined CD-specific mAb (GK1.5 anti-CD4). For the frequency ranges of the phenotyped cells, six different MNC samples from different hamsters were used for the analyses, but not all antibodies were tested at the same time (n = 3 or 4). This is why the% frequencies of HAB2A, HAL 26A and HAT19A have different ranges, in spite of recognising the same molecule. Nd = not determined. These mabs are listed as they are available for further characterisation by the research community.
